# Localised Kikuchi–Fujimoto Disease With Mediastinal Lymphadenopathy: A Case Initially Mimicking Malignant Lymphoma on Endobronchial Ultrasound‐Guided Transbronchial Needle Aspiration Cytology

**DOI:** 10.1002/rcr2.70521

**Published:** 2026-02-24

**Authors:** Kenta Nishiyama, Tomohiko Hasegawa, Tomohiko Ishimine, Risa Nagae, Taiga Shimabukuro, Mao Nishiyama, Natsumi Fukuzato, Yoshiaki Murayama, Kazunori Tamaki, Osamu Kakazu, Morio Ohta

**Affiliations:** ^1^ Department of Respiratory Medicine Nakagami Hospital Okinawa Japan; ^2^ First Department of Internal Medicine, Division of Infectious, Respiratory, and Digestive Medicine Graduate School of Medicine, University of the Ryukyus Ginowan Japan; ^3^ Department of Thoracic Surgery Nakagami Hospital Okinawa Japan

**Keywords:** EBUS‐TBNA, histiocytic necrotising lymphadenitis, Kikuchi–Fujimoto disease, malignant lymphoma, mediastinal lymphadenopathy

## Abstract

Kikuchi–Fujimoto disease (KFD), also known as histiocytic necrotising lymphadenitis, is a benign, self‐limiting disorder that primarily affects young women and is typically characterised by fever and cervical lymphadenopathy. However, isolated mediastinal lymphadenopathy without cervical involvement is rare. We report the case of a 37‐year‐old woman who presented with persistent fever and mediastinal lymphadenopathy. Endobronchial ultrasound‐guided transbronchial needle aspiration (EBUS‐TBNA) cytology of the lymph nodes revealed necrosis and atypical lymphoid cells, highly suggestive of malignant lymphoma. Owing to diagnostic difficulties, video‐assisted thoracoscopic excision was performed to obtain a definitive diagnosis. Histopathological examination confirmed KFD with massive necrosis and histiocytic infiltration. The patient underwent symptomatic treatment and remained under long‐term follow‐up. This case highlights the rarity of mediastinal‐limited KFD and the diagnostic pitfall of EBUS‐TBNA cytology, emphasising that surgical biopsy is critical for an accurate diagnosis when malignancy is suspected in this atypical presentation.

## Graphical abstract

We report a rare case of isolated mediastinal Kikuchi–Fujimoto disease (KFD) in a 37‐year‐old woman, which was initially misinterpreted as malignant lymphoma based on EBUS‐TBNA cytology. Definitive diagnosis was achieved through video‐assisted thoracoscopic surgery, revealing characteristic massive necrosis and histiocytic infiltration. This case highlights the diagnostic pitfalls of EBUS‐TBNA in atypical KFD presentations and underscores the necessity of surgical biopsy for an accurate diagnosis.

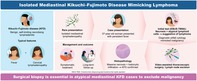

## 1 Introduction

Histiocytic necrotising lymphadenitis, also known as Kikuchi–Fujimoto disease (KFD), is a rare, self‐limiting, and benign syndrome first described in 1972. KFD predominantly affects young women and is classically characterised by fever and cervical lymphadenopathy that usually resolves spontaneously [1, 2]. However, the disease is rarely confined only to mediastinal lymph nodes without peripheral lymph node swelling in the neck, axilla or groin [2, 3, 4]. This atypical clinical presentation, consisting purely of fever and mediastinal lymphadenopathy, poses a significant diagnostic challenge, owing to the difficulty in differentiating it from serious diseases such as malignant lymphoma, tuberculosis and sarcoidosis [1]. Isolated mediastinal KFD is extremely rare, with only a few cases reported in the literature [3, 4]. Moreover, a definitive diagnosis requires histopathological examination.

Herein, we report an unusual case of KFD confined to the mediastinum in a young woman.

## 2 Case Report

A 37‐year‐old woman presented with persistent high fever (body temperature, 39°C) and chills lasting for more than a week. The patient had a history of smoking 15 cigarettes per day. The physical examination revealed no peripheral lymph node swelling in the head, neck, axilla or inguinal regions.

**FIGURE 1:**
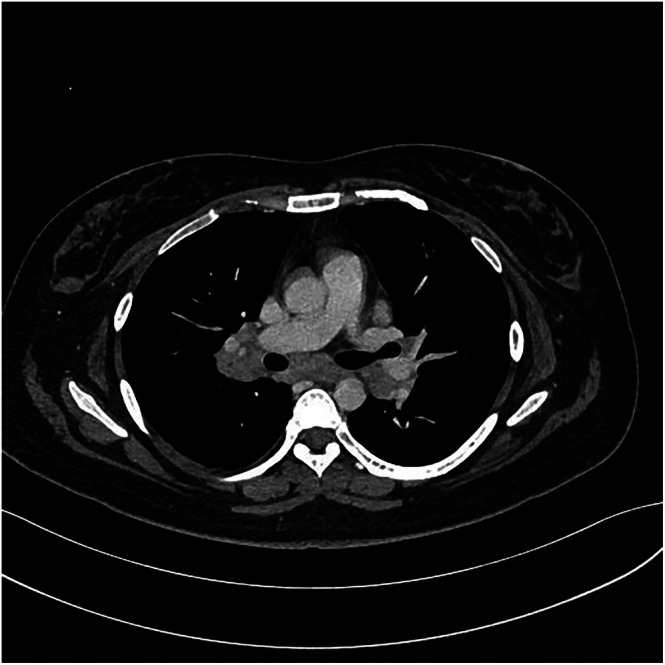
Contrast‐enhanced axial chest computed tomography image on admission. Soft tissue shadows are observed from the mediastinal to the bilateral hilar regions, indicating multiple lymphadenopathy. Findings are predominant on the right side, with some areas of low attenuation suggesting internal degeneration.

Initial laboratory findings revealed a highly elevated inflammatory response (C‐reactive protein, 11.78 mg/dL) and mild anaemia (haemoglobin, 8.6). Serum soluble IL‐2 receptor (sIL‐2R) levels were markedly elevated at 1767 U/mL. Contrast‐enhanced computed tomography confirmed mediastinal lymph node swelling (#4), with the largest node measuring approximately 3 × 2 cm (Figure 1). PET‐CT was not performed.

Endobronchial ultrasound‐guided transbronchial needle aspiration (EBUS‐TBNA) was performed on lymph node #4R. A 22‐gauge needle was used, with three passes. Rapid on‐site evaluation was not performed. Aspiration cytology revealed necrosis and atypical lymphocytes, suggestive of malignant lymphoma. Flow cytometry identified no abnormal phenotypes. Naproxen was administered, given the patient's deteriorating condition.

**FIGURE 2:**
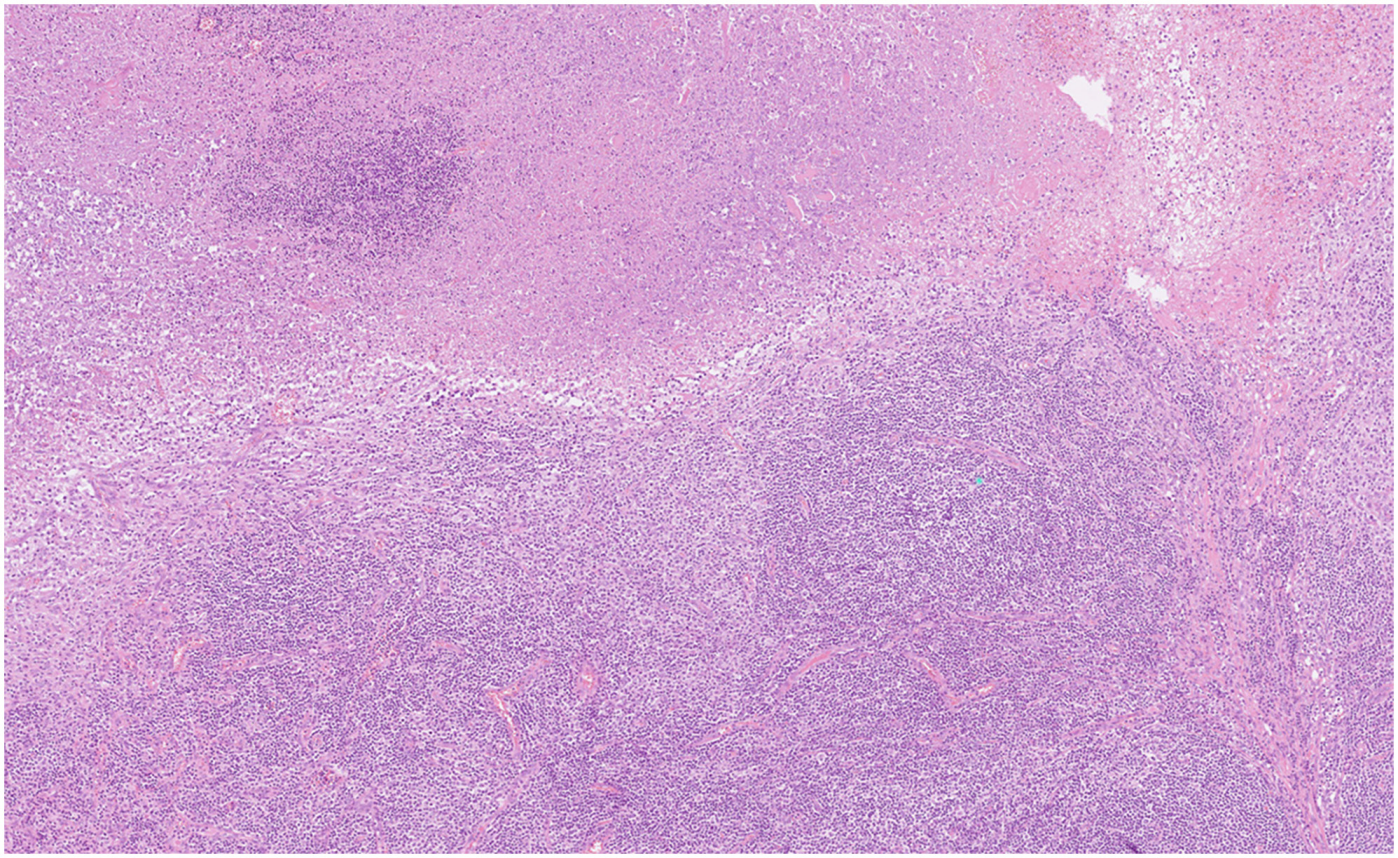
Histopathological examination of the resected mediastinal lymph nodes (haematoxylin and eosin staining, 100× magnification). The lymph node specimen shows extensive (or massive) necrosis scattered throughout the sample. The necrotic foci are filled with karyorrhectic debris (nuclear fragments/apoptotic bodies) and surrounded by numerous histiocytes, including foamy cells and apoptotic cells. Crucially, the specimen shows no definite granuloma formation, multinucleated cells (giant cells), neutrophils or malignant lymphoma cells/tumour cells. These findings are typical of histiocytic necrotising lymphadenitis (Kikuchi–Fujimoto disease).

Considering the strong suspicion of malignancy based on cytology and diagnostic difficulty, video‐assisted thoracoscopic resection of the right upper mediastinal lymph node (#4) was performed the following week to obtain adequate tissue. Histopathological examination confirmed the diagnosis of KFD, revealing extensive necrosis and histiocytic infiltration but no tumour cells or granulomatous findings (Figure 2). Immunohistochemical (IHC) analysis of the surgical specimen showed a reactive pattern (CD20, CD3, BCL2, kappa and lambda). IHC staining also revealed the presence of CD123‐positive plasmacytoid dendritic cells and scattered myeloperoxidase‐positive histiocytes, which are features consistent with KFD. Regarding the differential diagnosis, tuberculosis was ruled out by negative mycobacterial cultures and negative QuantiFERON‐TB Gold (QFT) 3G test results; however, tuberculosis polymerase chain reaction testing was not performed. Sarcoidosis was also excluded based on normal angiotensin‐converting enzyme levels and the absence of granulomas on histological examination. The symptoms improved with symptomatic treatment. Specifically, the patient was treated with naproxen (300 mg/day, divided into three doses) for 14 days. At the one‐year follow‐up, the patient exhibited a good general condition. Chest radiography performed 1 month postoperatively confirmed the absence of apparent lymphadenopathy.

## 3 Discussion

The present case highlights two critical points concerning the management of mediastinal lymphadenopathy: the rarity of isolated mediastinal KFD and the diagnostic limitations of EBUS‐TBNA cytology.

First, KFD presented as isolated mediastinal lymphadenopathy without cervical lesions [3, 4]. As KFD typically involves the cervical lymph nodes [1, 2], the absence of this hallmark finding can cause nonspecific symptoms, such as persistent fever and mediastinal lymphadenopathy (with elevated sIL‐2R), thus mimicking malignant lymphoma [2]. Moreover, as it is rarely confined to this specific site, it is often not considered early in the differential diagnosis of unexplained fever and mediastinal lymphadenopathy [1]. Second, this case highlights the significant diagnostic pitfalls of minimally invasive cytology techniques, such as EBUS‐TBNA. Although EBUS‐TBNA is highly effective for mediastinal lymphadenopathy related to many causes, it may have limitations in rare diseases like KFD where architectural assessment is critical for a definitive diagnosis. Newer ancillary techniques, such as EBUS‐guided forceps biopsy and EBUS‐guided cryobiopsy, may serve as useful alternatives and could potentially have avoided the need for surgical biopsy in this case. The initial cytology, which revealed necrosis and atypical lymphocytes, was a false finding that suggested malignant lymphoma. Notably, this is a recognised problem in KFD pathology, where extensive necrosis and nuclear fragmentation (nuclear collapse) frequently cause atypical lymphocytes in small cytology specimens to be misinterpreted as malignant tumour cells [5]. Specifically, studies focusing on fine‐needle aspiration cytology for KFD revealed a high false‐positive rate for malignancy (37.5%) [5].

Although EBUS‐TBNA is useful, it is generally considered insufficient for the definitive exclusion of lymphoma or sarcoidosis, owing to its small sample size [2, 4]. Furthermore, fine‐needle aspiration cytology techniques are inherently inadequate for achieving tissue‐based diagnoses, such as for KFD, sarcoidosis and lymphoma, compared with surgical biopsy [4, 5]. Therefore, when clinical symptoms are atypical and EBUS‐TBNA results are inconclusive or suggestive of malignancy, surgical biopsy, such as thoracoscopic resection or mediastinoscopy, is essential to avoid unnecessary aggressive treatments, such as chemotherapy [5]. Mediastinoscopy is a safe and effective method for obtaining definitive histological specimens in patients with isolated mediastinal lymphadenopathy and can confirm benign diseases such as KFD, which accounted for 2% of the diagnoses in a previous study [4].

In conclusion, isolated mediastinal KFD is a rare condition that is often misdiagnosed as malignancy owing to misleading EBUS‐TBNA cytology findings. This case highlights the need for a definitive surgical histological diagnosis when the possibility of malignancy cannot be ruled out in patients presenting with fever and isolated mediastinal lymphadenopathy.

## Author Contributions

All persons listed as authors qualify for authorship as detailed in the author guidelines. The authors collectively affirm that all authors were involved in the preparation and final approval of this manuscript and contributed significantly to the conception or design and the acquisition of data, analysis and interpretation of data for the case report.

## Funding

The authors have nothing to report.

## Consent

The authors declare that written informed consent was obtained for the publication of this manuscript and accompanying images using the consent form provided by the Journal.

## Conflicts of Interest

The authors declare no conflicts of interest.

## Data Availability

Data sharing not applicable to this article as no datasets were generated or analysed during the current study.
